# The association between serum copper concentration and prevalence of diabetes among US adults with hypertension (NHANES 2011–2016)

**DOI:** 10.1111/jcmm.18270

**Published:** 2024-04-03

**Authors:** Kaiming Wu, Lixia Chen, Yanyan Kong, Jian‐Feng Zhuo, Qiu Sun, Jianfei Chang

**Affiliations:** ^1^ Department of Chinese Medicine The Second Affiliated Hospital of Guangdong Medical University Guangdong China; ^2^ Department of Chinese Medicine Qingdao West Coast New Area People's Hospital Qingdao China; ^3^ Rehabilitation Medicine Department Qingdao West Coast New Area People's Hospital Qingdao China; ^4^ Geriatrics Department The Second Affiliated Hospital of Guangzhou University of Chinese Medicine Guangzhou China; ^5^ Surgery Teaching and Research Office Heilongjiang University of Chinese Medicine Harbin China

**Keywords:** copper, cross‐sectional study, diabetes, hypertension, NHANES

## Abstract

The objective of this study was to examine the association between the serum copper concentration and the prevalence of diabetes among US adults with hypertension using the data from the National Health and Nutrition Examination Survey (NHANES). The study population was selected from adults aged over 20 years old in the three survey cycles of NHANES from 2011 to 2016. Logistic regression model analyses were applied to determine the independent risky effect of copper to the prevalence of diabetes. Also, a restricted cubic spline (RCS) model was performed to explore the potential nonlinear association between serum copper concentration and the prevalence of diabetes. A total of 1786 subjects (742 cases and 1044 controls) were included, and 924 were men (51.7%), and 742 (41.5%) were diabetic. Compared with non‐diabetic individuals, the concentration of serum copper in diabetic patients with hypertension was higher. After adjusting for age, sex, race, education, marital status, body mass index (BMI), family poverty income ratio (PIR), smoking, alcohol drinking, physical activity, systolic blood pressure (SBP), diastolic blood pressure (DBP), and hyperlipidemia, the highest quartile of serum copper concentration significantly increased the risk of diabetes as compared with the lowest quartile (OR: 1.38, 95% CI: 1.01–1.92, *p*_trend_ = 0.036). The results of RCS analysis showed significant non‐linear relationship between serum copper concentration and prevalence of diabetes (*p*‐non‐linear = 0.010). This study finds that serum copper concentration are significantly associated with risk of diabetes in hypertensive patients, which suggests copper as an important risk factor of diabetes development.

## 1 INTRODUCTION

Diabetes mellitus, as a chronic non‐communicable disease, has a high epidemic intensity worldwide.^1^ The disease is characterized by hyperglycemia and is mainly caused by defective insulin secretion or impaired insulin bioactivity.^2^ The metabolic dysfunction may cause serious damage to many organs in the body, and is an important cause of premature death in the population.^3^, ^4^ According to estimates, the global prevalence of diabetes among people aged 20–79 years will be 10.5% (536.6 million people) in 2021, and is expected to rise to 12.2% (783.2 million people) in 2045.^5^ It is currently recognized that the aetiology of type 2 diabetes mellitus (T2DM) is closely related to genetic factors, environmental factors, immune factors and unhealthy lifestyle.^6^, ^7^, ^8^, ^9^ Absolute or relative insufficiency of insulin secretion and low insulin utilization efficiency were the main causes and mechanisms of T2DM.^10^ Considering the complexity of the pathogenesis of diabetes, it is particularly important to find some of the factors that influence the onset of the disease. Recently, there has been an increased emphasis on studying the effect of trace elements on the risk of diabetes.^11^, ^12^, ^13^

Copper is an essential mineral element for the human body which can participate in physiological processes such as erythropoiesis, immune function and resistance to oxidative stress damage, energy production, glucose metabolism and neuropeptide synthesis.^14^, ^15^ However, in recent years, a large number of studies have found that high exposure to copper may be associated with an elevated risk of developing a variety of chronic diseases, such as coronary heart disease, stroke and Alzheimer's disease.^16^, ^17^, ^18^, ^19^ Notably, the association between copper and diabetes has been widely recognized. A recent meta‐analysis by Qiu et al. found that diabetic patients carried higher levels of copper than healthy individuals.^20^ However, in Samadi's study, they concluded that copper levels may not have a significant impact on the association between diabetes.^21^ Hypertension and diabetes frequently co‐occur, posing a compounded risk for cardiovascular and metabolic complications. Considering that previous studies have reported that hypertensive patients have more than twice the risk of developing new‐onset diabetes than healthy individuals,^22^ and the controversy that exists in previous studies,^20^, ^21^ the objective of this study was to examine the association between the serum copper concentration and the prevalence of diabetes among US adults with hypertension using the data from the National Health and Nutrition Examination Survey (NHANES). By focusing on this specific demographic, the research seeks to uncover novel insights that could lead to more targeted and effective strategies for managing and preventing diabetes in hypertensive patients.

## 2 METHODS

### 2.1 Study design and population

For this cross‐sectional research, the publicly available data from the 2011 to 2016 National Health and Nutritional Examination Surveys (NHANES) was obtained. In brief, the NHANES is a series of representative cross‐sectional survey conducted in the United States by the Centers for Disease Control and Prevention (CDC) in order to provide information on nutrition and health.^23^, ^24^ Notably, stratified multistage sampling design was used to get a representative sample of the US general population. The research team was consisted with well‐trained health investigators, medical technicians and doctors. Interviews were first conducted with participants at home to collect background information such as medical history, family history, socio‐demographic, and so forth. Afterwards, they went to a Mobile Examination Center (MEC) to collect other relevant data, including anthropometric measurements, laboratory measurements, blood pressure, and so forth.

**FIGURE 1:**
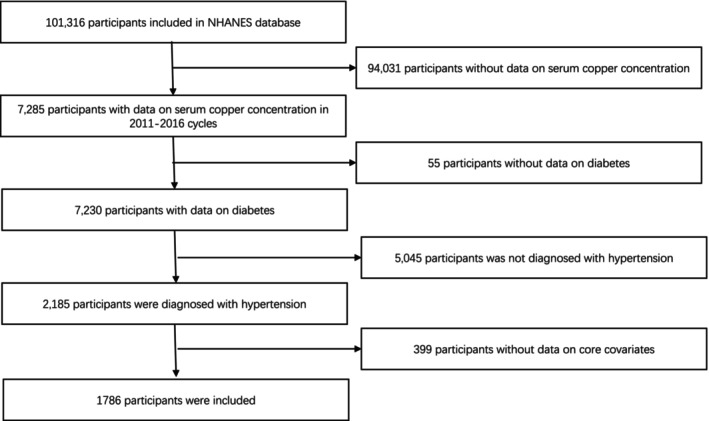
Flow chart.

Specifically, the present study used data from NHANES 2011 to 2016 cycle. It is important to note that the serum copper level measurements were only available for the years 2011–2016. Due to this data availability constraint, our analysis was limited to this time period. Subsequent years could not be included in our analysis as NHANES does not provide serum copper level data beyond 2016. The exclusion criteria were depicted as follows: (1) participants without data on serum copper concentration; (2) participants without data on diabetes; (3) CVD can be assessed by standardized questionnaires; (4) participants were not diagnosed with hypertension; (5) participants without data on core covariates. The detailed selection flow chart of included participants is presented in Figure 1.

### 2.2 Measurements of serum copper concentration

Serum copper was tested using inductively coupled plasma dynamic reaction cell mass spectrometry at the Environmental Health Sciences Laboratory of the National Center for Environmental Health of the Centers for Disease Control and Prevention, in accordance with extensive quality control procedures. In the binary logistic regression models, serum copper was categorized into four groups, according to the tertiles of the detected values.

### 2.3 Assessment of diabetes and hypertension

Diagnosis of diabetes mellitus is based on (1) symptoms of diabetes mellitus combined with a random blood glucose ≥11.1 mmol/L or (2) fasting blood glucose ≥7.0 mmol/L or (3) a positive oral glucose tolerance test.^25^ The criteria for diagnosing hypertension are as follows (1) self‐reported diagnosis of hypertension. (2) Subjects are taking antihypertensive medication. (3) Systolic blood pressure (SBP) over 130 mmHg or diastolic blood pressure (DBP) over 90 mmHg.^26^

### 2.4 Other variables

Covariates included sociodemographic, behavioural and health characteristics that were a priori considered potential confounders. Sociodemographic variables included age groups (20–39 years, 40–59 years, and ≥ 60 years), sex (male and female), race (Mexican American, non‐Hispanic Black, non‐Hispanic White and other race), education level (lower than high school, high school or equivalent and college or above), marital status (married/cohabiting, widowed/divorced/separated and never married), family poverty income (<1.3, 1.3–3.5 and >3.5). Behavioural characteristics consisted of smoking status (no or yes), alcohol consumption (no or yes), physical activity (no, moderate or vigorous). Health factors consisted of BMI (normal, overweight or obese) and hyperlipidemia (no or yes).

### 2.5 Statistical analysis

The relationship between serum copper and the risk of diabetes was determined using the using R4. 1. 3 software. Due to the skewed distribution of serum copper concentrations, the data were logarithmically (Ln) transformed. The demographic characteristics of the diabetic and non‐diabetic groups were described by the composition ratio (%), and the chi‐squared test was used to compare the differences between the two groups. Continuous variables were compared between groups using the Student *t*‐test or Mann–Whitney *U*‐test based on the normality of the distribution.

In this study, we utilized logistic regression analysis due to its effectiveness in handling binary outcome variables. Logistic regression is particularly advantageous for estimating the odds ratios (ORs), allowing us to understand the strength and direction of the association between serum copper levels and diabetes risk, while adjusting for potential confounders. So, serum copper levels were categorized into four groups (Q1 ~ Q4) according to quartiles, and logistic regression was performed with group Q1 as the reference group. Model 1 was a crude model, model 2 was adjusted for gender and age, and model 3 was complementally adjusted for race, education, marital status, PIR, BMI, smoking, alcohol drinking, physical activity, SBP, DBP, and hyperlipidemia.

Additionally, we employed restricted cubic splines (RCS) to explore potential non‐linear relationships between serum copper levels and diabetes risk. RCS is an advanced statistical technique that enables the modelling of complex, non‐linear relationships without assuming a specific functional form. By using RCS, we can better capture and illustrate any potential thresholds or curve patterns in the relationship between serum copper levels and diabetes risk, which might be overlooked in linear analyses. The dose–response association was finally calculated by RCS in fully adjusted model 3.

## 3 RESULTS

### 3.1 Characteristics of study participants

A total of 1786 hypertensive patients (Figure 1), including 924 men and 862 women, were enrolled in this study.742 of the participants were diagnosed with diabetes, accounting for 41.5% of the total. Compared with the non‐diabetic population, the diabetic population had lower education and income levels, higher BMI (*p* < 0.05), and higher proportions of older adults, alcohol drinkers and hyperlipidemia (*p* < 0.05). But there were no significant differences between the diabetic and non‐diabetic populations in gender and the proportion of smokers (*p* > 0.05). More detailed information was shown in Table 1.

**TABLE 1:** The characteristics of the study participants.

Variables	Total	Non‐DM	DM	*p*‐value
*N*	1786	1044	742	
Age, *n* (%)				
20–39	221 (12.37)	172 (16.48)	49 (6.60)	**<0.001**
40–59	571 (31.97)	354 (33.91)	217 (29.25)	
≥ 60	994 (55.66)	518 (49.62)	476 (64.15)	
Sex, *n* (%)				
Male	924 (51.74)	524 (50.19)	400 (53.91)	0.133
Female	862 (48.26)	520 (49.81)	342 (46.09)	
Ethnicity, *n* (%)				
Mexican American	209 (11.70)	99 (9.48)	110 (14.82)	**0.006**
Non‐Hispanic Black	469 (26.26)	277 (26.53)	192 (25.88)	
Non‐Hispanic White	750 (41.99)	457 (43.77)	293 (39.49)	
Other race	358 (20.04)	211 (20.21)	147 (19.81)	
Education, *n* (%)				
Lower than high school	453 (25.36)	215 (20.59)	238 (32.08)	**<0.001**
High school or equivalent	413 (23.12)	241 (23.08)	172 (23.18)	
College or above	920 (51.51)	588 (56.32)	332 (44.74)	
Marital status, *n* (%)				
Married/cohabiting	1021 (57.17)	610 (58.43)	411 (55.39)	**0.027**
Widowed/divorced/separated	544 (30.46)	294 (28.16)	250 (33.69)	
Never married	221 (12.37)	140 (13.41)	81 (10.92)	
BMI, *n* (%)				
Normal	258 (14.45)	188 (18.01)	70 (9.43)	**<0.001**
Overweight	444 (24.86)	281 (26.92)	163 (21.97)	
Obesity	1084 (60.69)	575 (55.08)	509 (68.60)	
PIR, *n* (%)				
<1.3	610 (34.15)	333 (31.90)	277 (37.33)	**0.002**
1–3.5	675 (37.79)	386 (36.97)	289 (38.95)	
>3.5	501 (28.05)	325 (31.13)	176 (23.72)	
Smoking status, *n* (%)				
No	890 (49.83)	523 (50.10)	367 (49.46)	0.829
Yes	896 (50.17)	521 (49.90)	375 (50.54)	
Alcohol consumption, *n* (%)				
No	261 (14.61)	134 (12.84)	127 (17.12)	**0.014**
Yes	1525 (85.39)	910 (87.16)	615 (82.88)	
Physical activity, *n* (%)				
No	726 (40.65)	385 (36.88)	341 (45.96)	**<0.001**
Moderate	578 (32.36)	342 (32.76)	236 (31.81)	
Vigorous	482 (26.99)	317 (30.36)	165 (22.24)	
Hyperlipidemia, *n* (%)				
No	347 (19.43)	235 (22.51)	112 (15.09)	**<0.001**
Yes	1439 (80.57)	809 (77.49)	630 (84.91)	
lnCu (mean [SD])	2.923 (0.217)	2.913 (0.209)	2.937 (0.228)	**0.021**

*Note*: The bold values means statistical significance.

### 3.2 The association between serum copper concentration and prevalence of diabetes among adults with hypertension

We investigated the relationship between serum copper levels and risk of diabetes using multiple logistic regression models. In model 1, without adjusting for any variables, it was found that higher level of serum copper was not associated with higher risk of diabetes, but the trend test was statistically significant (*p* for trend<0.05). In model 2, after adjusting for gender, age, it was found that higher serum copper concentration was positively associated with diabetes, and the trend test was statistically significant (*p* for trend<0.05). Compared with Q1, the OR of the highest serum copper levels group (Q4) was 1.49 (95% CI: 1.11–2.01). After adjusting all the covariate factors (including age, sex, race, education, marital status, PIR, BMI, smoking, alcohol drinking, physical activity, SBP, DBP and hyperlipidemia). It was found that the positive association between higher copper level and diabetes remained stable, and the trend test had statistical significance (*p* for trend<0.05). When taking the lowest quartile (Q1) as a reference, the level of serum copper concentration in the highest quartile (Q4) was associated with increased prevalence of diabetes (OR: 1.38, 95% CI: 1.00, 1.91).

In the total populations, the results of the univariate logistic regression model suggested positive association between ln‐transformed serum copper concentration and diabetes (OR: 1.67, 95% CI: 1.08, 2.60). In model 2, after adjusted for age, sex, the OR of diabetes was significantly associated with ln‐transformed serum copper concentration (OR: 2.30, 95% CI: 1.41, 3.79). Moreover, the result of model 3 (further adjusted for race, education, marital status, PIR, BMI, smoking, alcohol drinking, physical activity, SBP, DBP, and hyperlipidemia) suggested that ln‐transformed serum copper concentration was significantly associated with the increased occurrence of diabetes. The OR of the model and its 95% confidence interval (CI) were 2.09 (1.22, 3.62). The effect value of the model can be interpreted as a corresponding 109% increase in the probability of developing diabetes with increasing 1 unit of ln‐transformed serum copper concentration. More detailed results were presented in Table 2.

**TABLE 2:** The association between serum copper levels and prevalence of diabetes in 1786 US adults with hypertension by logistic regression analyses.

Serum copper levels, μg/dL	*N*	No. of case (%)	Model 1	Model 2	Model 3
			OR (95% CI)	OR (95% CI)	OR (95% CI)
ln‐transformed (continuous variables)	1786	742	**1.67 (1.08, 2.60)**	**2.30 (1.41, 3.79)**	**2.09 (1.22, 3.62)**
Q1 [3.88,16.16]	447	176 (39.4)	Reference	Reference	Reference
Q2 (16.16,18.56]	447	173 (38.7)	0.97 (0.74, 1.27)	0.96 (0.73, 1.26)	0.99 (0.74, 1.33)
Q3 (18.56,21.34]	448	191 (42.6)	1.14 (0.88, 1.49)	1.26 (0.95, 1.68)	1.15 (0.85, 1.56)
Q4 (21.34,41.61]	444	202 (35.5)	1.29 (0.99, 1.68)	**1.49 (1.11, 2.01)**	**1.38 (1.00, 1.91)**
*p* for trend			**0.033**	**0.002**	**0.036**

*Note*: **Model 1** was crude model. **Model 2** was adjusted for potential confounders, including age and sex. **Model 3** was additionally adjusted for race, education, marital status, PIR, BMI, smoking, alcohol drinking, physical activity, SBP, DBP, and hyperlipidemia. The bold values mean statistical significance.

**FIGURE 2:**
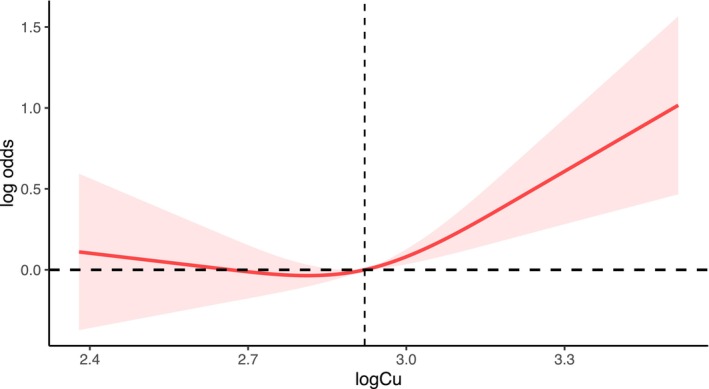
The restricted cubic spline (RCS) plot.

Besides, we used RCS curves to further elucidate the relationship between serum copper concentration and diabetes after adjusting for all covariates. As shown in Figure 2, the potential non‐linear association between serum copper concentration and the prevalence of diabetes was investigated.

### 3.3 Subgroup analyses

To explore whether there are differences in the association between serum copper concentration and the prevalence of diabetes in different subgroups. We performed logistic regression analyses stratified by characteristics such as age, gender, and so forth. The results of the multivariate logistic regression model showed that there was a significant positive correlation between serum levels of copper and diabetes only in women, the trend test was statistically significant (*p* for trend<0.05). However, no statistical correlation was found between in males. Moreover, the results of the multivariate logistic regression model showed that only the Q4 group of serum levels of copper and diabetes were found to have a positive relationship in the 40–59 age group, and trend tests were statistically significant (*p* for trend<0.05). Compared with Q1, the OR of the highest exposure level group (Q4) was 1.83 (95% CI: 1.01–3.34). Notably, Table 3 demonstrated that the positive association between the highest serum copper concentration category and occurrence of diabetes appeared stronger among non‐Hispanic Black, participants received education of college or above, obesity and drinkers. The ORs of these groups both had an obvious decreasing trend (All *p* for trend <0.05).

**TABLE 3:** The results of subgroup analyses.

	Tertile 1 OR (95% CI)	Tertile 2 OR (95% CI)	Tertile 3 OR (95% CI)	Tertile 4 OR (95% CI)	*p* for trend
Age					
20–39	Reference	1.71 (0.56, 5.27)	1.73 (0.63, 4.91)	2.46 (0.66, 9.41)	0.191
40–59	Reference	1.12 (0.65,1.94)	1.68 (0.94, 3.04)	**1.83 (1.01, 3.34)**	**0.030**
≥60	Reference	0.84 (0.57, 1.23)	0.87 (0.58, 1.30)	1.08 (0.71, 1.65)	0.696
Sex					
Male	Reference	1.15 (0.81, 1.62)	1.37 (0.92, 2.05)	1.15 (0.70, 1.88)	0.250
Female	Reference	0.78 (0.44, 1.39)	0.95 (0.56, 1.63)	1.38 (0.82, 2.35)	**0.032**
Ethnicity					
Mexican American	Reference	0.48 (0.19, 1.16)	0.50 (0.18, 1.33)	0.81 (0.28, 2.30)	0.720
Non‐Hispanic Black	Reference	1.38 (0.68, 2.83)	**1.96 (1.00, 3.94)**	**2.14 (1.08, 4.33)**	**0.022**
Non‐Hispanic White	Reference	1.10 (0.70, 1.73)	1.12 (0.70, 1.80)	1.40 (0.83, 2.36)	0.234
Other race	Reference	0.79 (0.42, 1.51)	1.06 (0.53, 2.11)	1.12 (0.52, 2.42)	0.695
Education					
Lower than high school	Reference	0.82 (0.45, 1.49)	1.02 (0.54, 1.93)	1.09 (0.58, 2.06)	0.619
High school or equivalent	Reference	0.84 (0.44, 1.61)	1.13 (0.59, 2.19)	1.39 (0.68, 2.85)	0.282
College or above	Reference	1.17 (0.78, 1.76)	1.21 (0.79, 1.86)	**1.59 (1.00, 2.52)**	0.061
Marital status					
Married/cohabiting	Reference	0.91 (0.63, 1.32)	1.08 (0.73, 1.61)	1.36 (0.86, 2.14)	0.180
Widowed/divorced/separated	Reference	1.12 (0.62, 2.01)	1.25 (0.71, 2.22)	1.41 (0.80, 2.49)	0.212
Never married	Reference	1.18 (0.40, 3.46)	1.25 (0.42, 3.71)	1.84 (0.61, 5.78)	0.285
BMI					
Normal	Reference	0.66 (0.26, 1.62)	0.75 (0.26, 2.06)	1.12 (0.41, 3.07)	0.789
Overweight	Reference	0.76 (0.43, 1.33)	0.80 (0.43, 1.49)	1.19 (0.60, 2.36)	0.741
Obesity	Reference	1.24 (0.84, 1.83)	**1.49 (1.01, 2.21)**	**1.76 (1.16, 2.69)**	**0.007**
PIR					
<1.3	Reference	1.15 (0.65, 2.05)	1.33 (0.76, 2.36)	1.53 (0.87, 2.73)	0.123
1–3.5	Reference	1.04 (0.65, 1.67)	1.46 (0.89, 2.39)	1.64 (0.97, 2.81)	**0.035**
>3.5	Reference	0.84 (0.49, 1.44)	0.86 (0.48, 1.54)	1.06 (0.53, 2.08)	0.998
Smoking status					
No	Reference	0.98 (0.64, 1.49)	1.09 (0.71, 1.67)	1.42 (0.89, 2.28)	0.145
Yes	Reference	1.05 (0.69, 1.60)	1.32 (0.85, 2.06)	1.39 (0.88, 2.20)	0.107
Alcohol consumption					
No	Reference	0.55 (0.24, 1.23)	0.70 (0.29, 1.68)	0.80 (0.33, 1.95)	0.814
Yes	Reference	1.06 (0.77, 1.46)	1.23 (0.88, 1.71)	**1.46 (1.03, 2.09)**	**0.026**
Physical activity					
No	Reference	0.71 (0.43, 1.17)	1.06 (0.64, 1.74)	1.10 (0.66, 1.85)	0.346
Moderate	Reference	1.51 (0.90, 2.54)	1.03 (0.60, 1.76)	**1.81 (1.01, 3.27)**	0.142
Vigorous	Reference	0.91 (0.52, 1.58)	1.60 (0.86, 2.99)	1.67 (0.84, 3.32)	0.070
Hyperlipidemia					
No	Reference	0.74 (0.34, 1.56)	0.63 (0.28, 1.40)	1.47 (0.65, 3.34)	0.471
Yes	Reference	1.03 (0.74, 1.42)	1.25 (0.89, 1.74)	1.36 (0.95, 1.95)	0.054

*Note*: The results were adjusted for age, sex, ethnicity, education levels, marital status, body mass index (BMI), family poverty income ratio (PIR), smoking status, alcohol consumption, physical activity, SBP, DBP, and hyperlipidemia. The bold values means statistical significance.

## 4 DISCUSSION

Diabetes is a common endocrine system disease. It is a metabolic syndrome characterized by prolonged hyperglycemia due to defects in insulin secretion and action resulting in metabolic disorders of carbohydrates, fats and proteins.^27^ The incidence of diabetes mellitus has been on the rise in recent years, and its chronic complications have a serious impact on the quality of patients.^28^ The pathogenesis of diabetes mellitus mainly includes genetic, environmental and immunologic factors.^29^ Studies have showed that oxidative stress causes pancreatic islet cell dysfunction and insulin resistance, which resulted in tissue cell damage.^30^

At the meantime, more and more studies are focusing on the role of trace elements in the development of diabetes. Although trace elements are found at extremely low levels in the body, they are involved in the composition of molecular structures such as hormones, nucleic acids and catalytic enzymes.^31^ Trace elements are closely related to the biological functions of molecules, and the abnormal levels can affect the metabolism and functions of tissues and cells.^32^, ^33^ For example, in a meta‐analysis of 22 cohort studies, Zhao et al. identified an inverse association between magnesium intake and diabetes.^34^ Also, Safarzad et al. found that lower serum zinc level is associated with higher fasting insulin in T2DM.^11^ Not only that, but the role of copper is coming into focus. High plasma copper concentrations have been reported to mediate the formation of damaging ROS, which are associated with insulin resistance.^35^, ^36^ A population‐based study reported an association between copper levels and hypertension, and Darroudi et al. found that serum copper levels within a certain range may be associated with elevated blood pressure.^37^ Given that the co‐occurrence of hypertension and diabetes was prevalent among the adults,^38^ this study aimed to explore the association between serum copper concentration and the prevalence of diabetes among hypertensive adults using the NHANES database.

This study offers a novel examination in the US hypertensive adults of the role of serum copper concentration regarding its association with diabetes and considering the confounding role of various factors. It is worth noting that non‐Hispanic Black, participants received education of college or above, obesity and drinkers were more likely to show such association. Before this study, there was a previous published article assessing the association between plasma copper concentration and diabetes in hypertensive Chinese adults.^39^ However, given the racial differences as well as environmental differences, it remains unclear whether the above conclusions are analogous among Americans. Therefore, our study is likely to have an important role to evaluate the influence of copper in adults of different regions.

A large population‐based prospective cohort study concluded that dietary intakes of copper was associated with a higher risk of diabetes in Japanese population.^40^ Considering the potential recall bias of dietary intake surveys and individual differences in copper intake, absorption and metabolism, more previous studies have measured copper levels in biological samples to reflect copper exposure and accumulation in human body. In Ekin's study, they found that copper levels were higher, in diabetic patients, than in the controls.^41^ Besides, a multicentre study by Raudenska demonstrated that copper was significantly increased in the diabetics.^42^ The conclusions of the above articles are consistent with the findings of this study. However, a case control study in Norway generated different conclusion. This might be related to the smaller sample size and the difference in the region.^43^

Our study's subgroup analysis reveals significant demographic variations in the association between serum copper levels and diabetes risk. This aspect of our research provides a deeper understanding of how this relationship may differ across various population segments. For instance, we found a notable positive correlation between high serum copper levels and diabetes risk among non‐Hispanic Black participants, those with a higher education level, and individuals classified as obese. These findings align with previous studies indicating the heightened vulnerability of specific demographic groups to metabolic disorders.

Previous research exploring the mechanism of influence of serum copper concentrations on diabetes provided multiple possible explanations for the study's findings. First, due to its redox properties, copper is able to catalyse the Fenton reaction and the Haber–Weiss reaction,^44^ both of which may generate large amounts of hydroxyl radicals and further lead to elevated levels of ROS in the body. This may directly cause macromolecular damage or indirectly lead to oxidative stress,^45^ resulting in abnormal insulin secretion and decreased β cell function decline.^46^ Second, Cu_2_+ interacts with pancreatic islet amyloid polypeptides to produce H_2_O_2_ and mediates the process involved in the aggregation of human pancreatic islet amyloid polypeptides into amyloidogenic fibrils,^47^ which is critical for the progressive degeneration of pancreatic islet cells in T2DM.

The advantages of this research included the first report of serum copper concentration being positively associated with the risk of diabetes, the evaluation of a dose–response association between serum copper concentration and diabetes in hypertensive adults in US, the presentation of stratified analyses. This study specifically examines hypertensive individuals, a group with a heightened risk of developing diabetes. By analysing the relationship between serum copper levels and diabetes risk within this particular demographic, the research provides crucial insights into the interplay between hypertension and metabolic disorders, which is essential for understanding the link between these common health issues. Moreover, the findings from this study could reveal serum copper as a potential marker for diabetes risk in hypertensive patients. This opens avenues for early intervention strategies that target copper levels, potentially aiding in the prevention or management of diabetes in this high‐risk group.

However, this cross‐sectional study also has other drawbacks because of its observational nature. First, the cause‐and‐effect relationship between serum copper concentration and the occurrence of diabetes cannot be well established from this study. Thus, further epidemiological cohort studies or controlled trials would be required to evaluate the relationship between copper and diabetes in hypertensive adults. Second, while this study offers valuable insights into the relationship between serum copper levels and diabetes risk in hypertensive individuals, it is important to acknowledge certain methodological considerations. Notably, the analysis was conducted without the application of NHANES' complex survey weighting. However, this approach allowed for a more straightforward analysis and interpretation of the data. Future studies might incorporate these weights to provide a more representative analysis of the national trends and associations. Third, recall bias is a concern in any study relying on self‐reported data, such as NHANES.

## 5 CONCLUSION

In summary, our results revealed that serum copper concentration may be associated with the risk of diabetes among US adults with hypertension. In the future, larger prospective cohort studies are warranted to explore such finding and to investigate causal inference. Longitudinal studies could provide deeper insights into how serum copper levels influence the progression from hypertension to diabetes.

## AUTHOR CONTRIBUTIONS

**Kaiming Wu:** Formal analysis (equal); investigation (equal). **Lixia Chen:** Writing – review and editing (equal). **Yanyan Kong:** Conceptualization (equal); methodology (equal). **Jian‐Feng Zhuo:** Writing – original draft (equal). **Qiu Sun:** Writing – review and editing (equal). **Jianfei Chang:** Funding acquisition (equal); methodology (equal); project administration (equal).

## CONFLICT OF INTEREST STATEMENT

The authors made no disclosures.

## Data Availability

Data available on request from the authors.
